# Genetic approach toward linkage of Iran 2012–2016 cholera outbreaks with 7th pandemic *Vibrio cholerae*

**DOI:** 10.1186/s12866-024-03185-9

**Published:** 2024-01-22

**Authors:** Fatemeh Jalalizadeh, Elisabeth Njamkepo, François-Xavier Weill, Forough Goodarzi, Marjan Rahnamaye-Farzami, Roghieh Sabourian, Bita Bakhshi

**Affiliations:** 1 Department of Bacteriology, Faculty of Medical Sciences, Tarbiat Modares University, Tehran, Iran; 2 Institut Pasteur, Université Paris Cité, Paris, France; 3 Research Center of Health Reference Laboratories, Tehran, Iran

**Keywords:** *Vibrio cholerae*, PFGE, MLST, Cholera, Iran, WGS

## Abstract

*Vibrio cholerae*, as a natural inhabitant of the marine environment is among the world-leading causes of diarrheal diseases. The present study aimed to investigate the genetic relatedness of Iran 2012–2016 V*. cholerae* outbreaks with 7th pandemic cholera and to further characterize the non-ST69/non-ST75 sequence types strains by whole-genome sequencing (WGS).

Twenty *V. cholerae* isolates related to 2012, 2013, 2015 and 2016 cholera outbreaks were studied by two genotyping methods – Pulsed-field Gel Electrophoresis (PFGE) and Multi-locus Sequence Typing (MLST)–and by antimicrobial susceptibility testing. Seven sequence types (STs) and sixteen pulsotypes were detected. Sequence type 69 was the most abundant ST confirming that most (65%, 13/20) of the studied isolates collected in Iran between 2012 and 2016 belonged to the 7th pandemic clone. All these ST69 isolates (except two) exhibited similar pulsotypes. ST75 was the second most abundant ST. It was identified in 2015 and 2016. ST438, ST178, ST579 and STs of 983 and 984 (as newfound STs) each were only detected in one isolate. All strains collected in 2016 appeared as distinct STs and pulsotypes indicative of probable different originations. All ST69 strains were resistant to nalidixic acid. Moreover, resistance to nalidixic acid, trimethoprim-sulfamethoxazole and tetracycline was only observed in strains of ST69. These properties propose the ST69 as a unique genotype derived from a separate lineage with distinct resistance properties. The circulation of *V. cholerae* ST69 and its traits in recent years in Iran proposes the 7th pandemic strains as the ongoing causes of cholera outbreaks in this country, although the role of ST75 as the probable upcoming dominant ST should not be ignored.

Genomic analysis of non-ST69/non-ST75 strains in this study showed ST579 is the most similar ST type to 7th pandemic sequence types, due to the presence of wild type-El Tor sequences of *tcpA* and VC-1319, VC-1320, VC-1577, VC-1578 genes (responsible for polymyxin resistance in El Tor biotype), the traits of *rstC* of RS1 phage in one strain of this ST type and the presence of VPI-1 and VSP-I islands in ST579 and ST178 strains. In silico analysis showed no significant presence of resistance genes/cassettes/plasmids within non-ST69/non-ST75 strains genomes. Overall, these data indicate the higher susceptibility of *V. cholerae* non-ST69/non-ST75 strains in comparison with more ubiquitous and more circulating ST69 and ST75 strains.

In conclusion, the occurrence of small outbreaks and sporadic cholera cases due to *V. cholerae* ST69 in recent years in Iran shows the 7th pandemic strains as the persistent causes of cholera outbreaks in this country, although the role of ST75 as the second most contributed ST should not be ignored. The occurrence of non-ST69/non-ST75 sequence types with some virulence factors characteristics in border provinces in recent years is noteworthy, and further studies together with surveillance efforts are expected to determine their likely route of transport.

**Supplementary Information:**

The online version contains supplementary material available at 10.1186/s12866-024-03185-9.

## Introduction

*Vibrio cholerae*, as a bacterial water and foodborne pathogen, causes significant morbidity and mortality worldwide. Cholera symptoms include dehydrating diarrhea, vomiting and abdominal cramps [1, 2]. *V. cholerae* is responsible for seven important pandemics of cholera since 1817 [3]. According to a World Health Organization (WHO) report, it has been evaluated that millions of cholera cases and tens of thousands of mortalities happen each year around the world [4]. Epidemiological and source tracking investigation of cholera outbreaks in human societies often requires typing of strains to identify the prevalence and relatedness of epidemic and endemic isolates. All these events emphasize on typing investigations to determine the source of outbreak and improve public health, and monitoring vaccination programs [5–7].

Different typing methods have been developed for outbreak surveillance of *V. cholerae* strains. The conventional phenotypic epidemiological tools including serotyping, phage typing and antibiogram are inadequate to provide standardized description of clonal definition of *V. cholerae* isolates involved in outbreaks. Different genotyping tools have also been developed for subspecies-level classification of *V. cholerae.* These methods include serotyping, multilocus sequence typing (MLST) [8], multilocus variable number of tandem repeats (VNTR) analysis (MLVA) [9], single-nucleotide polymorphism (SNP)- based approaches and Core genome MLST (cgMLST) in dealing with large data sets [10]. Despite some important limitations to each, these methods are progressively used individually or in combination, to provide the best approach to bacterial genetic relatedness.

Among genotyping methods, Pulsed-field Gel Electrophoresis (PFGE) has been selected as the gold standard in molecular epidemiology of small-scale outbreaks [11]. The power of discrimination and reproducibility of PFGE has made it a widely useful method for bacterial species genotyping [12]. Some limitations of PFGE include identical band patterns are not always guaranteeing homologous genetic content and the inability of gel-based methods to resolve similar sized bands which could be resolved in combination with other genotyping methods.

Multi-locus Sequence Typing (MLST) as a sequence-based method, investigates variations in seven housekeeping genes and provides information on population structure and evolution of bacterial species as well as their epidemiological significance. Furthermore, MLST provides comparable, portable and reproducible genotypic data which can be readily exchanged among laboratories [13–16].

Core genome MLST (cgMLST) which relies on whole-genome sequencing (WGS), although providing a superior scheme and more phylogenetic resolution than traditional MLST [10], there is a need to continue with MLST amplicon sequencing to allow some level of genomic epidemiology where access to second or third generation sequencing is limited, especially in dealing with large data sets in low-income and middle-income countries.

In global surveillance of pandemic-causing pathogens, such as *V. cholerae*, it is important to use an efficient and easy to use genotyping tool, with the potential to be applied to all *V. cholerae* strains around the world.

Combining PFGE and MLST analysis can provide higher resolution in characterization of *V. cholerae* isolates and their relationship to each other. In the present study, we investigated the genetic relatedness of *V. cholerae* strains collected from 2012–2016 cholera outbreaks in Iran using PFGE and MLST analysis. Moreover, the non-ST69/non-ST75 sequence types strains were further characterized using whole-genome sequencing (WGS).

## Materials and methods

### Ethics statement

This study protocol was approved by the Ethics Committee of Tarbiat Modares University (Code: IR.TMU.REC.1397.092). All patients completed a questionnaire and gave informed consent prior to the study.

### Bacterial strains

We studied 20 V*. cholerae* strains from stool sample of cholera patients in Iran in 2012 (*n* = 5), 2013 (*n* = 5), 2015 (*n* = 6) and 2016 (*n* = 4). These *V. cholerae* strains were selected from our collection containing 11 strains isolated in 2012 (⁓20% of total cholera cases in Iran this year), 33 in 2013 (⁓13% of total cholera cases in Iran this year), 6 in 2015 (⁓7% of total cholera cases in Iran this year) and 4 in 2016 (no data on total cholera cases). Due to high level of clonality among strains of 2012 and 2013 [17, 18], five representatives from each year were randomly selected and studied.

The isolates were confirmed by biochemical and molecular methods. After enrichment by alkaline peptone water, yellow colonies on thiosulfate citrate bile salts sucrose (TCBS agar) were isolated and tested for oxidize, Kligler iron slant agar reaction, arginine and esculin hydrolysis, Lysine and ornithine decarboxylase activity and growth in 0% NaCl [19, 20]. All twenty strains were characterized by polyclonal O1 and Ogawa and Inaba specific antisera (Bahar Afshan, Iran). Biotyping of isolates was performed by the Voges-Proskauer (VP) test and the sheep red blood cells Hemagglutination assay [21]. Molecular identification of *V. cholerae* isolates was performed by PCR with primers for targeting the 16S-23S rRNA intergenic region as previously described [22].

### Antimicrobial susceptibility testing

Antibiotic susceptibility was tested by disk diffusion method according to Clinical and Laboratory Standards Institute (CLSI-M45) guidelines [23, 24] with following antibiotics: ampicillin (10 µg), cefotaxime (30 µg), ceftazidime (30 µg), gentamicin (10 µg), tetracycline (30 µg), ciprofloxacin (5 µg), trimethoprim-sulfamethoxazole (1.23/23.75 µg), chloramphenicol (30 µg), azithromycin, erythromycin and nalidixic acid. All antimicrobial disks were purchased from Mast Group (Mast Group Ltd, UK). The minimum inhibitory concentrations (MICs) of ampicillin were determined by the agar dilution method. The MIC E-test strip (Liofilchem, Italy) was used for determination of MIC of azithromycin and erythromycin for all strains.

### Pulsed-field gel electrophoresis

PFGE was performed on 10 isolates from 2015 and 2016, based on the Standard Operating Procedure for PulseNet PFGE of *Vibrio cholerae* and *Vibrio parahaemolyticus* [25]. Briefly, Proteinase K (Bioneer, South Korea) and *Not*I restriction enzyme (Thermo Fisher Scientific, US) was used for digestion of the genomic DNA of bacterial cells. PFGE was performed with a CHEF Mapper system (BioRad) with a two-block program under the following conditions: The block 1 ranged from 2–10 s for 13 h and block 2 from 20–25 s for 6 h at 6.0 V/cm. *Salmonella* serotype Branderup H9812 was utilized as DNA molecular mass size marker. PFGE pattern of isolates 2012 and 2013 were available from our previous researches [17, 18]. PFGE banding patterns were analyzed by BioNumerics software (version 7.6.3; Applied Maths, Belgium). The dendrogram was generated using Dice similarity index and UPGMA method. The strains with more than > 30% similarity fell into major clusters, then types and subtypes were assigned according to criteria presented by Tenover et al. [26].

### Multi-locus Sequence Typing (MLST)

Genomic DNA was extracted with AccuPrep genomic extraction kit (Bioneer, Korea) for all amplification tests. Seven housekeeping genes (*adk, gyrB*, *metE, mdh, pntA, purM, pyrC*) were used for MLST analysis [8]. Amplification of genes was performed by primers that are shown in Table 1. Each PCR was performed with an initial denaturation at 95℃ for 3 min, denaturing at 95℃ for 30 s, extension at 72℃ for 1 min and a final extension at72℃ for 2 min repeated for 34 cycles. Annealing temperatures were adjusted according to melting temperature of each primer sets. PCR reactions were carried out in 25 µl total reaction volume containing 8 µl PCR-Master mix (Taq DNA Polymerase 2 × Master Mix RED, 1.5 mM MgCl2) (Ampliqon, Denmark), 2 µl template DNA, 1 μl of each of the primers (10 pmol), and 13 µl sterilized distilled water. The DNA purification and sanger sequencing were performed by Microsynth (Switzerland). Sequences were trimmed, placed in the right direction and aligned at the same start and stop positions by CLC Genomic Workbench (version 12.0; Qiagen) and BioNumerics softwares (version 7.6.3; Applied Maths, Belgium). The alleles were uploaded to PubMLST and assigning the alleles and sequence type of strains were defined according to PubMLST website (https://pubmlst.org/organisms/vibrio-cholerae).

**Table 1:** Primers used in this study

Gene	Gene product	Oligonucleotide sequence	Product size (bp)	Annealing	Reference
*adk*	adenylate kinase	CATCATTCTTCTCGGTGCTC	592	59℃	[8]
		AGTGCCGTCAAACTTCAGGTA			
*gyrB*	DNA gyrase subunit B	GTACGTTTCTGGCCTAGTGC	749	60℃	[8]
		GGGTCTTTTTCCTGACAATC			
*metE*	methionine synthase	CGGGTGACTTTGCTTGGT	827	58℃	[8]
		CAGATCGACTGGGCTGTG			
*mdh*	malate dehydrogenase	ATGAAAGTCGCTGTTATTGG	692	60℃	[8]
		GCCGCTTGGCCCATAGAAAG			
*pntA*	pyridine nucletide transhydrogenase	GGCCAGCCCAAAATCCT	758	59℃	[8]
		GATATTGCCGTCTTTTTCTT			
*purM*	phosphoribosylformylglycinamide cyclo-ligase	GGTGTCGATATTGATGCAGG	734	58.4℃	[8]
		GGAATGTTTTCCCAGAAGCC			
*pyrC*	Dihydroorotase	ATCATGCCTAACACGGTTCC	726	56℃	[8]
		TTCAAACACTTCGGCATA			
*ctxA/B*	Cholera toxin	TATGCCAAGAGGACAGAGTGAG	115	55℃	[27]
		AACATATCCATCATCGTGCCTAAC			
*tcpA*	Toxin co-regulated pilus subunit A	GGTCAGCCTTGGTAAGGTT	225	50℃	This study
		CAAGATCAGCGACAGCAG			

### Real-time PCR for toxin gene detection

Real-time PCR was used for detection of cholera toxin (*ctxA/ctxB* genes) and toxin co-regulated pilus (*tcpA* gene). Primer sequences are described in Table 1. The SYBR green Premix EX Taq mixture (Takara, Japan) was in a total volume of 20µL containing 0.8µL of each primer pair, 2µL of DNA sample, and 6.4µL of distilled water. RT- PCR cycling conditions were as follows: 95°C for 5 min and 40 cycles of 95°C for 15s, 60°C for 43 s.

### Whole-genome sequencing (WGS) and analysis of selected strains

Genomic DNA was extracted using AccuPrep genomic extraction kit (Bioneer, Korea). Whole genome sequencing was performed using the NovaSeq 6000 and HiSeq 2500 instruments (Illumina,San Diego, California, USA). DNA libraries were prepared using a Nextera DNA Flex Library Preparation Kit (Illumina), followed by 2 × 150 bp paired-end sequencing runs with a median coverage of 171 X (95– 628). Short-read sequence data were submitted in the European Nucleotide Archive (ENA) (http://www.ebi.ac.uk/ena) and the accession numbers are listed in Table 2.

**Table 2:** The non-ST69/non-ST75 strains characterized via genomic analysis

Isolate ID	Genome ID (accession no)/Database	Genomic coverage (X)	Country of isolation	Year of isolation	ST	Source of isolation	O-serogroup *(*in silico)	AMR phenotype	AMR gene	Plasmids	Genomic Islands	*ctxA/B*	*tcpA*	Reference
195	2016_1 s(ERR6323226)/(EBI-ENA)	195	Iran	2016	579	Human	O1	Pan susceptible	*qnrVC4*	/	VPI-1	/	wt	This study
2134	2134/PubMLST	/	India	2017	579	Human	O1	No data	*qnrVC4*	/	VPI-1	/	wt	PubMLST
2106	2106/ PubMLST	/	Morocco	2015	579	Human	O1	No data	*qnrVC4*	/	VPI-1	/	wt	PubMLST
295	CNRVC170253 (ERR6323228)/(EBI-ENA)	171	Iran	2016	178	Human	O1	Pan susceptible	/	/	VSP-I	/	/	This study
1884	1884/ PubMLST	/	Russia	2004	178	Human	O1	No data	/	/	VSP-I	/	/	PubMLST
1402	1402 /PubMLST	/	Ukraine	2016	178	Human	O1	No data	/	/	VSP-I	/	/	PubMLST
1403	1403/ PubMLST	/	Ukraine	2017	178	Human	O1	No data	/	/	VSP-I	/	/	PubMLST
491	2012_4 (ERR6323225)/(EBI-ENA)	628	Iran	2012	438	Human	O2	Pan susceptible	/	/	/	/	/	This study
391	CNRVC170247 (ERR6323227)/(EBI-ENA)	126	Iran	2012	983	Human	O7	Ampicillin	*bla*_CARB-7_/*catB9*	/	/	/	/	This study
495	CNRVC170254 (ERR6323229)/(EBI-ENA)	95	Iran	2016	984	Human	O1	Pan susceptible	/	/	/	/	/	This study

Pan susceptible: Susceptible to all antibiotics

wt: Wild-type *V. cholerae* O1 El Tor Inaba strain N16961

(/): Not detected

Short reads were first cleaned using FqCleanER version 3.0 (https://gitlab.pasteur.fr/GIPhy/fqCleanER) to eliminate adaptor sequences [28], correct sequencing errors [29], and discard low-quality short-reads. Assemblies were generated de novo by SPAdes version 3.15.0 [30] with default settings.

Genome assemblies were aligned with the reference genome of *Vibrio cholerae* O1 El Tor N16961 using Blast software (https://blast.ncbi.nlm.nih.gov). Different genetic markers were analyzed against reference sequences of the O-antigen biosynthetic gene clusters (O-AGCs), CTX prophage, the *ctxB* gene, the toxin co-regulated pilus (TCP) genes, the *Vibrio* pathogenicity islands 1/2, and the *Vibrio* seventh pandemic islands I/II (Table 3).

**Table 3:** The reference DNA sequences used in this study

Genomic sequence analyzed	Accession numbers (GenBank/DDBJ)
*Vibrio cholerae* O1 El Tor N16961	AE003852.1 and AE003853.1
CTX prophage	CTX-1, AE003852, coordinates 15666967–1573281 CTX-2, CP001486, coordinates 852233–858550
*ctxB* gene	(AE003852, coordinates 1566967–1567341)
toxin co-regulated pilus (TCP) genes	(AE003852, coordinates 890449–891123)
*Vibrio* pathogenicity island 1	(VPI-1, AE003852, coordinates 873242–914124)
*Vibrio* pathogenicity island 2	(VPI-2, AE003852, coordinates 1896092- 1952861)
*Vibrio* seventh pandemic island I	(VSP-I, AE003852, coordinates 175343–189380)
*Vibrio* seventh pandemic island II	(VSP-II, AE003852, coordinates 523156–550021)
O-AGC	rfb from O1 (LC594800), O2 (LC594901) and O7(LC594961)

The presence and type of genomic islands (GIs), acquired antibiotic resistance genes (ARGs) and plasmids were determined with VCGIDB (http://leb.snu.ac.kr/vcgidb/index) [31], ResFinder v4.0.1 (https://cge.cbs.dtu.dk/services/ResFinder/) [32] and PlasmidFinder v1.3 (https://cge.cbs.dtu.dk/services/PlasmidFinder/) [33].

### Additional genomic data

In July of 2021, only five genomic sequences from ST579 (*n* = 2) and ST178 (*n* = 3) strains were available via PubMLST (https://pubmlst.org/organisms/vibrio-cholerae). Fasta sequence files from available sequence data were downloaded and included in the analyses (Table 2).

## Results

### Identification and confirmation of *V. cholerae* isolates

Eighteen *V. cholerae* isolates were confirmed as O1 serogroup. One was O2 and the remaining isolate was O7. According to VP and the sheep red blood cells hemagglutination tests, 19 out of twenty isolates were identified as El Tor biotype and only one isolate (*V. cholerae* O2) showed phenotypic characteristics of classical biotype.

### Antimicrobial susceptibility testing

The antimicrobial susceptibility showed that all the isolates were susceptible to cefotaxime, ceftazidime, gentamicin, chloramphenicol and azithromycin. Resistance to trimethoprim-sulfamethoxazole, tetracycline and nalidixic acid was seen in 40% (8/20), 40% (8/20) and 65% (13/20) of isolates, respectively (Table 4). All strains except one (ID:391) were susceptible to ampicillin as determined by MIC method (Table 4).

**Table 4:** Antimicrobial resistance phenotypes of studied *V. cholerae* isolates

Isolate ID	Isolation	ST	Year of Isolation	*GM*	*CHL*	*AZM*	*ERY*	*AMP*	*TET*	*NAL*	*SXT*	*CIP*	CTX	CZA
	**Province**													
**191**	**Baluchistan**	**69**	**2012**	**S**	**S**	**S**	**S**	**S**	**R**	**R**	**R**	**S**	**S**	**S**
**391**	**Baluchistan**	**983**	**2012**	**S**	**S**	**S**	**I**	**R**	**S**	**S**	**S**	**S**	**S**	**S**
**491**	**Baluchistan**	**438**	**2012**	**S**	**S**	**S**	**S**	**S**	**S**	**S**	**S**	**S**	**S**	**S**
**891**	**Baluchistan**	**69**	**2012**	**S**	**S**	**S**	**S**	**S**	**R**	**R**	**R**	**S**	**S**	**S**
**991**	**Baluchistan**	**69**	**2012**	**S**	**S**	**S**	**S**	**S**	**R**	**R**	**R**	**S**	**S**	**S**
**192**	**Baluchistan**	**69**	**2013**	**S**	**S**	**S**	**S**	**S**	**R**	**R**	**R**	**S**	**S**	**S**
**292**	**Baluchistan**	**69**	**2013**	**S**	**S**	**S**	**S**	**S**	**R**	**R**	**R**	**S**	**S**	**S**
**392**	**Baluchistan**	**69**	**2013**	**S**	**S**	**S**	**S**	**S**	**R**	**R**	**R**	**S**	**S**	**S**
**492**	**Baluchistan**	**69**	**2013**	**S**	**S**	**S**	**S**	**S**	**R**	**R**	**R**	**S**	**S**	**S**
**592**	**Baluchistan**	**69**	**2013**	**S**	**S**	**S**	**S**	**S**	**R**	**R**	**R**	**S**	**S**	**S**
**194**	**Khuzestan**	**75**	**2015**	**S**	**S**	**S**	**S**	**S**	**S**	**S**	**S**	**S**	**S**	**S**
**894**	**Khorasan**	**69**	**2015**	**S**	**S**	**S**	**S**	**S**	**S**	**R**	**S**	**I**	**S**	**S**
**994**	**Khorasan**	**69**	**2015**	**S**	**S**	**S**	**S**	**S**	**S**	**R**	**S**	**S**	**S**	**S**
**104**	**Tehran**	**69**	**2015**	**S**	**S**	**S**	**S**	**S**	**S**	**R**	**S**	**I**	**S**	**S**
**114**	**Qazvin**	**69**	**2015**	**S**	**S**	**S**	**S**	**S**	**S**	**R**	**S**	**S**	**S**	**S**
**124**	**Qom**	**69**	**2015**	**S**	**S**	**S**	**S**	**S**	**S**	**R**	**S**	**I**	**S**	**S**
**195**	**Bushehr**	**579**	**2016**	**S**	**S**	**S**	**S**	**S**	**S**	**S**	**S**	**S**	**S**	**S**
**295**	**Bushehr**	**178**	**2016**	**S**	**S**	**S**	**I**	**S**	**S**	**S**	**S**	**S**	**S**	**S**
**395**	**Khuzestan**	**75**	**2016**	**S**	**S**	**S**	**S**	**S**	**S**	**S**	**S**	**S**	**S**	**S**
**495**	**Khuzestan**	**984**	**2016**	**S**	**S**	**S**	**S**	**S**	**S**	**S**	**S**	**S**	**S**	**S**

*Abbreviations*: *GM* gentamicin, *CHL* chloramphenicol, *AZM* azithromycin, *ERY* erythromycin, *AMP* ampicillin, *TET* tetracycline, *NAL* nalidixic acid, *SXT* trimethoprim-sulfamethoxazole, *CIP* ciprofloxacin, *CTX* cefotaxime, *CZA* ceftazidime, *S* susceptible, *R* resistant

All ST69 strains were resistant to nalidixic acid. Moreover, resistance to nalidixic acid, trimethoprim-sulfamethoxazole and tetracycline was only observed in ST69 strains.

### MLST analysis

**Fig. 1:**
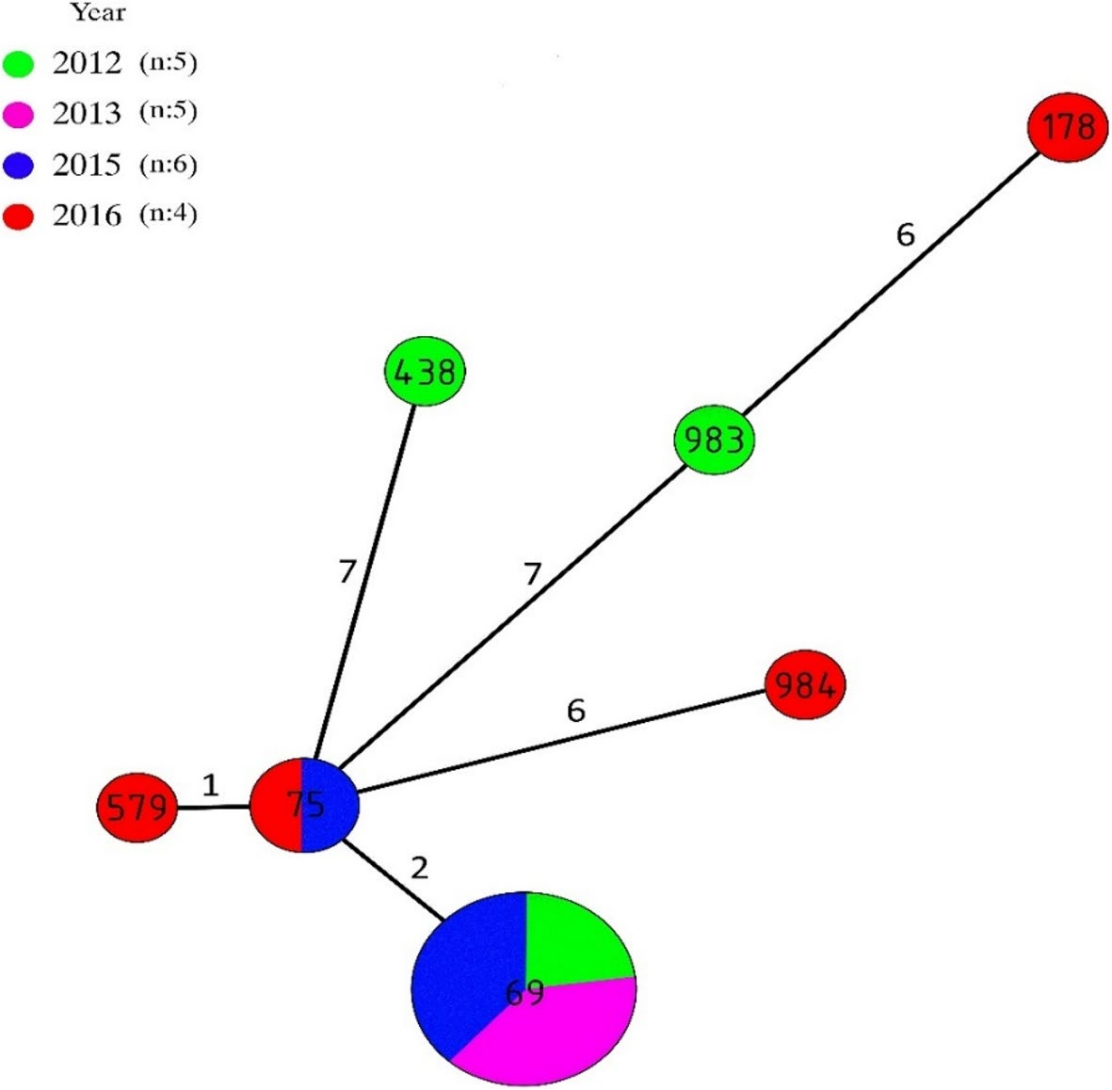
The minimum spanning tree based on STs of V. cholerae strains. The size of nodes represents STs numbers and distance between nodes represents the locus differences. The digits next to the lines between the two nodes represent the number of different loci

All amplicon sequences were submitted and assigned by PubMLST. In total, seven sequence types (STs) were identified among 20 V*. cholerae* isolates in this study. The ST69 was the most abundant ST and was identified in 13 (65%) isolates (from 2012, 2013 and 2015). This ST is the predominant ST among seventh pandemic *V. cholerae* El Tor isolates, which has also been reported from at least 37 countries (https://pubmlst.org/organisms/vibrio-cholerae). ST75, was the second most prevalent ST identified in 10% of our isolates (from 2015 and 2016). The remaining 5 STs, including 2 newfound STs (983 and 984), each were detected in a single isolate. Based on MLST data, the genetic relatedness of these seven STs was assessed by a minimum spanning tree (Fig. 1). Accordingly, ST75 is the most closely related ST to ST69, and ST579 is a single-locus variant (SLV) of ST75.

**Fig. 2:**
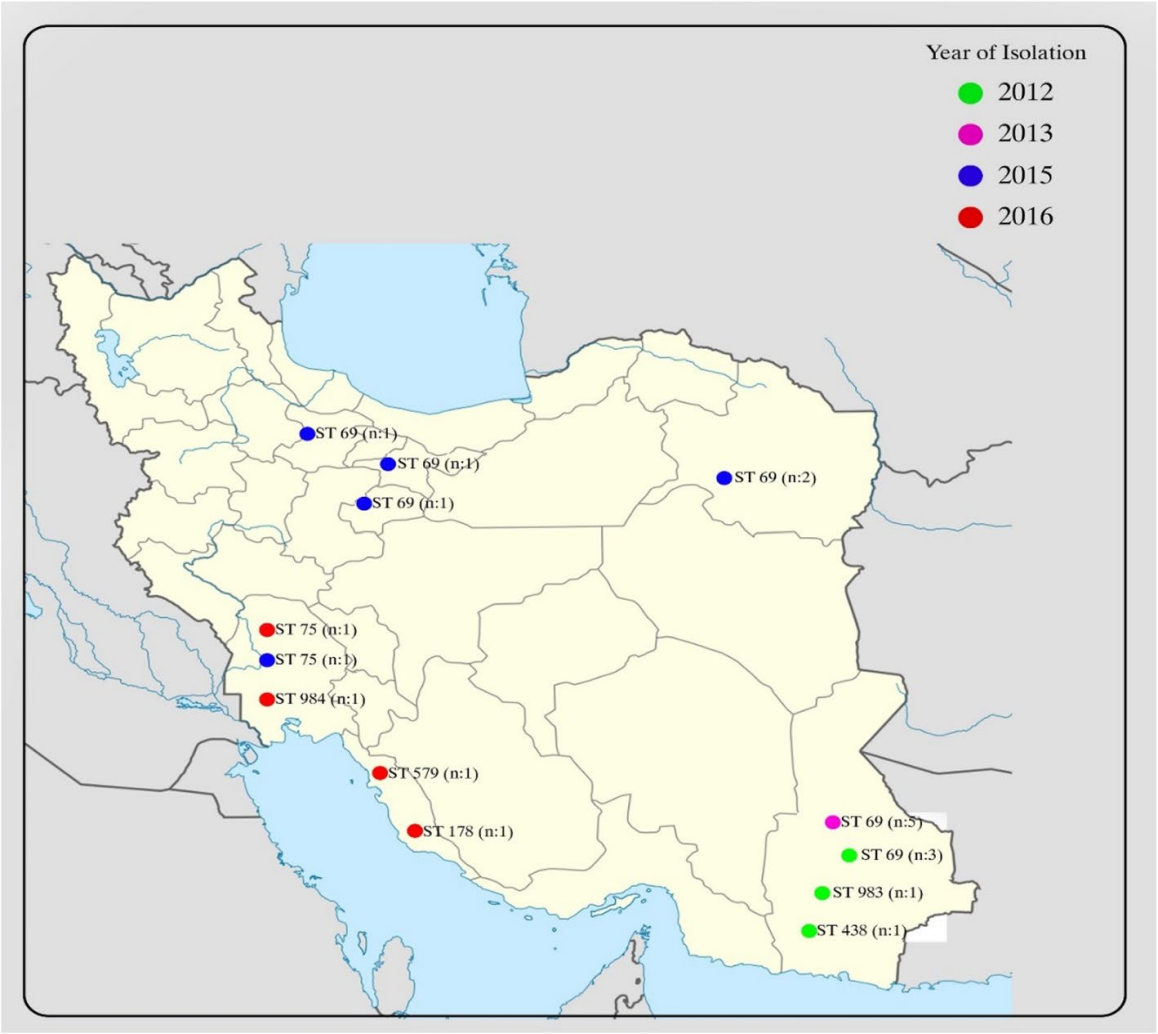
The distribution of V. cholerae strains in this study according to the sequence types and year of isolation. Sequence types detected in each province are shown by ST number. Members of each ST are defined within parenthesis. Isolates belonging to each year are depicted by a distinct color. As depicted in picture, strains of non-ST69/non-ST75 were mainly isolated from border provinces in south of Iran. Free map was obtained from Wikimedia commons (https://commons.wikimedia.org/wiki/File:Iran_location_map.svg)

**Fig. 3:**
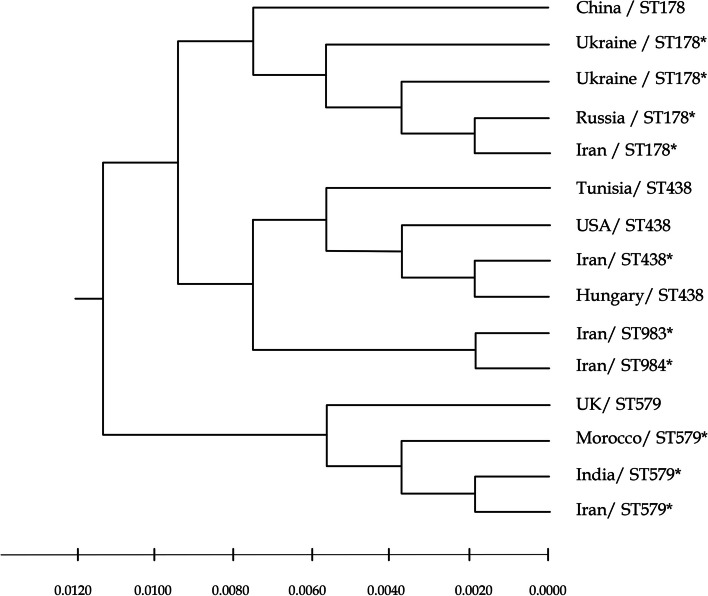
A phylogenetic tree of all V. cholerae isolates with ST178, ST438, ST983, ST984 and ST579 sequence types which have been deposited in PubMLST (https://pubmlst.org/organisms/vibrio-cholerae). The tree was constructed based on MLST profiles using MEGA software (version 11) based on the maximum composite likelihood and UPGMA. * V. cholerae isolates with WGS data used in this study

Members of all STs were defined as O1/El Tor serogroup/biotype, except for ST983 (O7/El Tor) and ST438 (O2/Classical). The STs, their allele types and geographical location of *V. cholerae* isolates is summarized in Table 5 and Fig. 2. As depicted in Fig. 2, ST69 strains were more widely distributed around the country, while ST75 strains were restricted to South West Iran. Moreover, strains from 2012 and 2013 were isolated from South East Iran, the Baluchistan province which shares a common border with Afghanistan and proposes their probable import from this neighbor country. It should be noted that cholera cases in border provinces in south of Iran were solely of non-ST69/non-ST75 (Fig. 2), raising the hypothesis of their importing from our neighboring countries, all of those countries encounter cholera epidemics annually. A phylogenetic tree of *V. cholerae* isolates with ST 178, 438, 983, 984 and 579 from PubMLST is shown in Fig. 3. The tree is reflecting the relatedness of non-ST69/non-ST75 according to MLST data. Accordingly, the ST983 and ST984 are the two most closely related STs, which together with ST438 comprising a distinct cluster from ST178 and ST579.

**Table 5:** Allele types and ST numbers of *V. cholerae* strains in relation to their virulence genes content

Isolate ID	Year of Isolation	*adk*	*gyrB*	*mdh*	*metE*	*pntA*	*purM*	*pyrC*	ST	Serogroup	ctxA/B	*tcpA*
191	2012	7	11	4	37	12	1	20	69	O1	+	+
391	2012	1	153	68	243	2	43	1	983	O7	-	-
491	2012	2	44	11	64	3	8	43	438	O2	-	-
891	2012	7	11	4	37	12	1	20	69	O1	+	+
991	2012	7	11	4	37	12	1	20	69	O1	+	+
192	2013	7	11	4	37	12	1	20	69	O1	+	+
292	2013	7	11	4	37	12	1	20	69	O1	+	+
392	2013	7	11	4	37	12	1	20	69	O1	+	+
492	2013	7	11	4	37	12	1	20	69	O1	+	+
592	2013	7	11	4	37	12	1	20	69	O1	+	+
194	2015	7	2	4	37	12	1	38	75	O1	-	+
894	2015	7	11	4	37	12	1	20	69	O1	+	+
994	2015	7	11	4	37	12	1	20	69	O1	+	+
104	2015	7	11	4	37	12	1	20	69	O1	+	+
114	2015	7	11	4	37	12	1	20	69	O1	+	+
124	2015	7	11	4	37	12	1	20	69	O1	+	+
195	2016	7	2	4	13	12	1	38	579	O1	-	+
295	2016	1	31	56	99	70	48	13	178	O1	-	-
395	2016	7	2	4	37	12	1	38	75	O1	-	+
495	2016	2	38	161	230	69	1	259	984	O1	-	-

### Detection of toxin genes

Real-time PCR showed the presence of *ctxA/B* gene in 13/20 (65%) strains (Table 5). In fact, all ST69 strains harbored the *ctxA*/*B* genes while, all non-ST69 strains lacked the toxin genes. The *tcpA* gene was detected in 16/20 (75%) strains (strains of ST69, ST75 and ST579 genotypes).

### PFGE analysis

**Fig. 4:**
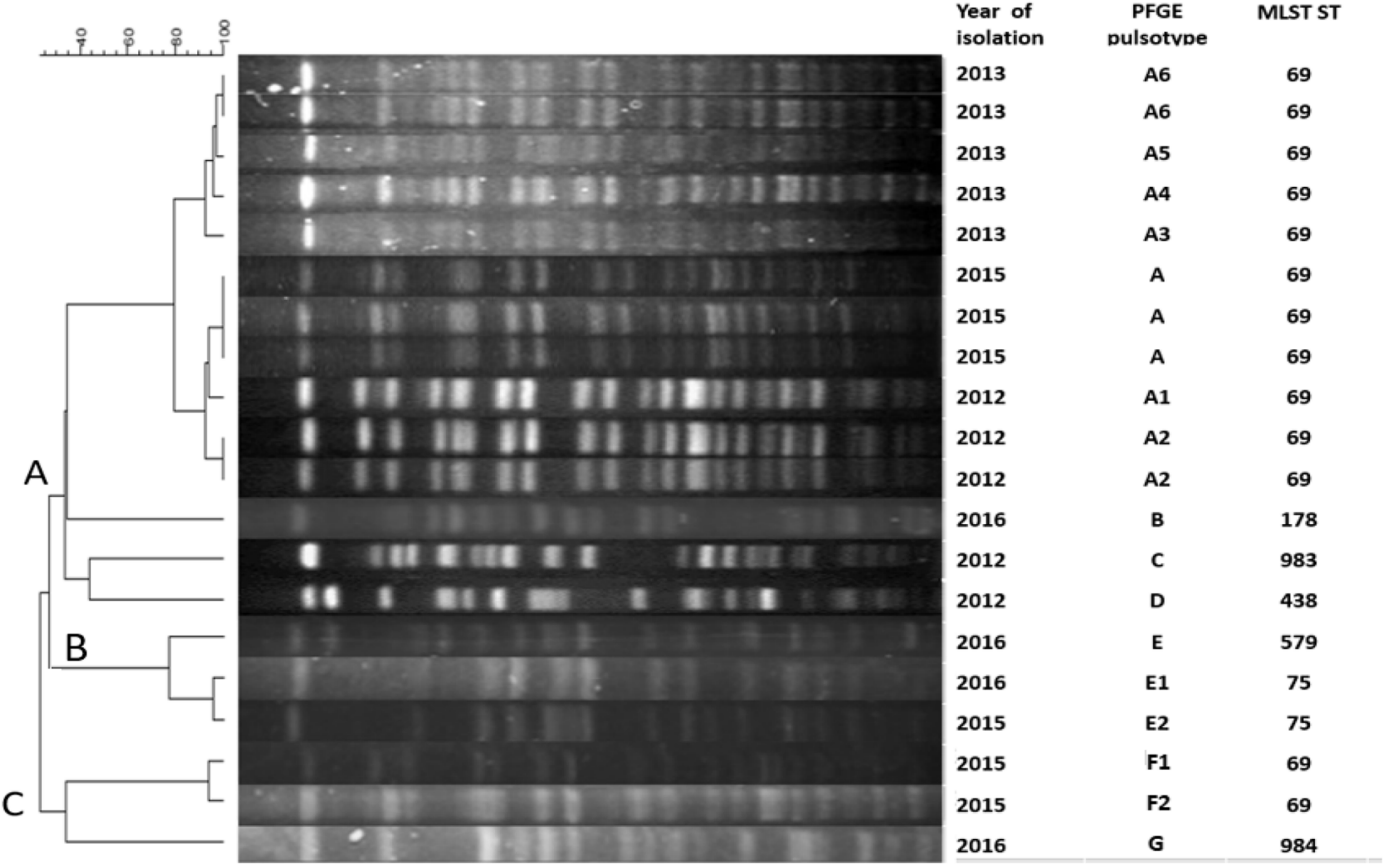
Pulsed-field Gel Electrophoresis (PFGE) patterns of V. cholerae strains in this study. Each strain is shown in relation to year of isolation, pulsotype name and related sequence type (ST). V. cholerae strains are classified to three clusters **A**, **B** and **C** based on a similarity cutoff of 30%. Moreover, each pulsotype is assigned a distinct letter (A to G) and subtypes of each pulsotype are defined with numerical subscripts. * Full-length blots/gels associated to PFGE patterns of V. cholerae strains in current study are presented in Supplementary Fig. 1

Seven different pulsotype letters (A-G) were assigned to 20 V*. cholerae* strains which were analyzed based on PFGE method. Eleven isolates (from 2012, 2013 and 2015) showed identical or very similar pulsotypes, which was considered as the dominant pulsotype (A) of this study. All the isolates with pulsotype A uniformly belonged to ST69 (Fig. 4).

### Whole-genome sequence analyses of ST178, ST983, ST984, ST438 and ST579 strains

Except for well-known ST69 and ST75 types, little information is available on genomic data and gene content of strains belonging to other sequence types. Therefore, non-ST69/non-ST75 strains in this study were subjected to WGS to make a conceptual analysis and comparison (Table 2).

WGS analysis of non-ST69/non-ST75 strains including 5 from Iran (this study) and 5 from PubMLST showed that none of them harbored *ctxA* or *ctxB* genes, while a wild type-El Tor *tcpA* was present only in strains of ST579 (Table 2). The O1-antigen gene (*rfbO1*) was present in all strains of ST579, ST178 and ST984 indicative of O1 serogroup specificity.

Wild type-El Tor sequences of VC-1319, VC-1320, VC-1577, VC-1578 genes (responsible for polymyxin resistance in El Tor biotype) was detected in all strains of ST579 (from Iran, India and Morocco). These genes are supposed to be altered in Classical strains which restore their susceptibility to polymyxin B [34, 35].

Moreover, the *rstC* gene, the signature of RS1 phage, was also present in one genome of ST579 (ID:2106 from pubMLST). This means that ST579 genomes are prone for acquisition of CTX page and its satellite RS1, although its stability is not well guaranteed.

The beta-lactamase *bla*_CARB-7_ gene was detected in an ampicillin-resistant ST983 strain from Iran (ID:391).

The *catB9* gene was also found in this ST983 strain, which was phenotypically susceptible to chloramphenicol. As indicated in previous studies, this gene is a silent chloramphenicol acetyl transferase gene within a super integron of *V. cholerae* which is not considered as indicative of resistance when identified in whole-genome sequences of *V. cholerae* El Tor [36, 37]. The *qnrVC4* gene (Quinolone resistance pentapeptide repeat protein QnrVC4) was detected in all three ST579 strains. This gene has also been found in quinolone-susceptible strains of ST75 (SLV of ST579) in South Africa [38]. This gene, different alleles of which are located in a chromosomal super-integron, is not necessarily related to resistance as indicated here and in several previous studies [39, 40].

## Discussion

As an endemic disease, cholera outbreaks are annually reported from Iran; However, cross-border cases from neighbor countries including Pakistan, Afghanistan and Iraq, are also observed in some provinces. To our knowledge, approximately 400 cholera cases have been reported from Iran during 2012–2016 (mainly May–November) [41, 42]. This prompted us to undertake the genetic characterization of recent cholera outbreaks and better understanding of endemic cholera.

The significant contribution of current study was the investigation of prevalent STs in accordance with related pulsotypes in Iran during 2012–2016. The 7th pandemic clone, ST69, was revealed as the predominant circulating clone in Iran during 2012–2016 and all strains in this ST appeared as pulsotype A or F and their subtypes (A1 to A6) (F1 and F2). Subtypes of each pulsotype are indicative of 1–2 genetic event, i.e., a point mutation or an insertion or deletion of DNA and are considered as minor differences from predominant pulsotype [26], consistent with ST69 sub-lineages [10].

Interestingly, all isolates of the present study with pulsotype A, uniformly fell in ST69. Moreover, the two ST75 isolates together with a single locus variant of ST75 (ST579) were assigned to a separate pulsotype letter (pulsotype E). This may validate PFGE as a mirror of MLST typing and a powerful epidemiological tool in *V. cholerae* investigations. The global circulation of ST69 might lead to some genetic events (mutations, deletion, insertion) within the genome content of *V. cholerae* strains which may be reflected in minor differences in PFGE patterns. By careful consideration of studies reporting PFGE patterns of ST69 strains, these minor variations in PFGE patterns of ST69 strains could be clearly elicited [43]. This means that strains belonging to ST69 might show minor differences in pulsotype, despite of identical ST type. ST69 as cholera 7th pandemic strain, has been reported from Asia, Africa, America and Europe [43–46].

**Fig. 5:**
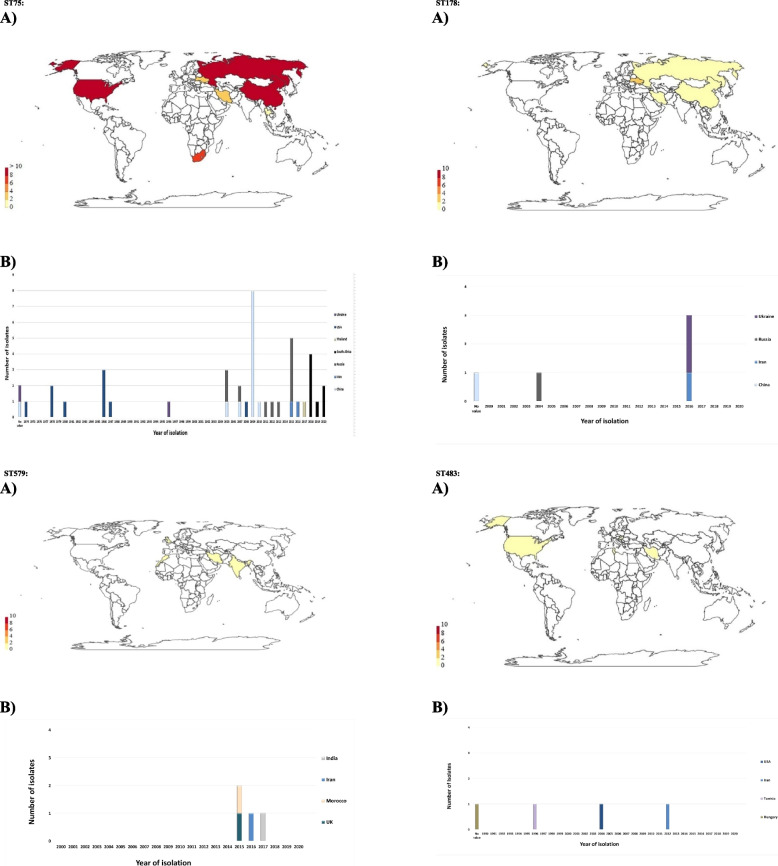
Geographic distribution of ST75, ST178, ST579 and ST438 around the world (PubMLST website, July 2021). A) According to country and number of isolates. B) According to identification year and number of isolates. * No value indicates that the year of isolation of V. cholerae strains is unknown

ST75, as the second most prevalent ST in our study, has also been identified in seven countries including Iran, China, Russia, USA, Ukraine and Thailand and South Africa (according to pubMLST database), but its frequency did not display considerable changes among years except for 2009, which affected Thailand and became more prevalent than the ST69 clone afterward (Fig. 5). Subsequently, related ST75 strains have emerged in several countries and is now somehow widespread in Asia and America continents [8, 47, 48]. In China, ST75 was the most prevalent sequence type among non-7th pandemic clone strains in Zhejiang Province, its emergence and potential spread draw significant attention due its probable threat to public health [47]. Moreover, ST75 recently emerged and became more prevalent than the pandemic clone in South Africa during 2018–2020 [38]. The occurrence of two isolates of ST75 in 2015 and 2016 is noteworthy, indicating the probable beginning of sporadic cholera due to ST75 in Iran; however, it should be considered that they may be endemic to the region but not previously detected by the surveillance system. The advent of ST75 in provinces bordering the Persian Gulf, like some other countries which were affected by US indigenous Gulf Coast-like strains, can enhance the probability of ST75 overtaking in similar regions in global view.

ST438 which was detected among our isolates of 2012, was previously confirmed in three countries, Hungary, Tunisia and the US. This ST is, for the first time, reported from an Asian country (Fig. 5). The single Iranian strain (ID: 491) belonging to this ST type, appeared as classical biotype according to phenotypic analyses. Due to little available data on sequence types of non-O1/non-O139 strains and considerable diversity among their population, careful consideration should be undertaken in interpretations.

Large-scale cholera outbreaks have decreased in recent years in Iran due to improvements in hygienic level and lifestyle of people in this country, although small outbreaks and sporadic cholera cases mainly of non-ST69/non-ST75 strains is still an ongoing concern in bordering provinces. Genomic analysis of non-ST69/non-ST75 strains in this study showed i) the presence of wild type sequence of *tcpA* in ST579 strains, ii) the wild type-El Tor sequence of VC-1319, VC-1320, VC-1577, VC-1578 genes (responsible for polymyxin resistance in El Tor biotype) in strains of ST579, iii) the *rstC* gene, indicative of RS1 phage in one of the ST579 strains, and iv) the presence of VPI-1 and VSP-I islands in ST579 and ST178 strains. These data, all together, indicate that ST579 has more virulence and pathogenic potential compared with other sequence types under study (ST178, ST983, ST984 and ST438). In silico analysis of resistance genes and cassettes showed that i) none of the known antibiotic resistance genes or cassettes were present among the non-ST69/non-ST75 strains under study, ii) SXT constin responsible for resistance to several antimicrobials was not detected, and iii) no plasmid was detected among the isolates. Overall, these data indicate the high susceptibility of *V. cholerae* non-ST69/non-ST75 strains in comparison with more ubiquitous and more globally ST69 and ST75 strains.

In conclusion, the occurrence of *V. cholerae* strains of non-ST69/non-ST75 sequence types with some traits of virulence factors in recent years in Iran is noteworthy. Extensive studies together with surveillance efforts are expected to determine their likely route of transport. The occurrence of these STs, although still susceptible to current antimicrobial agents, is important because they may gradually change in antibiotic resistance gene content. Moreover, the circulation of *V. cholerae* ST69 in recent years in Iran shows the 7th pandemic strains as the persistent causes of cholera outbreaks in this country, although the role of ST75 as the second most contributed ST should not be ignored.

### Supplementary Information

**Additional file 1.**

## Data Availability

The confirmed DNA sequences of MLST analysis were deposited in GenBank under accession numbers MN026296-MN026329. Short-read sequence data from WGS were deposited in European Nucleotide Archive (https://www.ebi.ac.uk/) under accession numbers ERR6323225- ERR6323229. All datasets of the current study are available within article or can be obtained from corresponding with no restriction.

## References

[1] Pierce NF, Sack RB, Mitra RC, Banwell JG, Brigham KL, Fedson DS (1969). Replacement of water and electrolyte losses in cholera by an oral glucose—electrolyte solution. Ann Intern Med.

[2] Hirschhorn N, Kinzie JL, Sachar DB, Northrup RS, Taylor JO, Ahmad SZ (1968). Decrease in net stool output in cholera during intestinal perfusion with glucose-containing solutions. N Engl J Med.

[3] Lacey SW (1995). Cholera: calamitous past, ominous future. Clin Infect Dis.

[4] Ali M, Lopez AL, You YA, Kim YE, Sah B, Maskery B (2012). The global burden of cholera. Bull World Health Organ.

[5] Foley SL, Lynne AM, Nayak R (2009). Molecular typing methodologies for microbial source tracking and epidemiological investigations of gram-negative bacterial foodborne pathogens. Infect Genet Evol.

[6] Olive DM, Bean P (1999). Principles and applications of methods for DNA-based typing of microbial organisms. J Clin Microbiol.

[7] Rahaman M, Islam T, Colwell RR, Alam M (2015). Molecular tools in understanding the evolution of Vibrio cholerae. Front Microbiol.

[8] Octavia S, Salim A, Kurniawan J, Lam C, Leung Q, Ahsan S (2013). Population structure and evolution of non-O1/non-O139 Vibrio cholerae by multilocus sequence typing. PLoS ONE.

[9] Olsen JS, Aarskaug T, Skogan G, Fykse EM, Ellingsen AB, Blatny JM (2009). Evaluation of a highly discriminating multiplex multi-locus variable-number of tandem-repeats (MLVA) analysis for Vibrio cholerae. J Microbiol Methods.

[10] Liang KY, Orata FD, Islam MT, Nasreen T, Alam M, Tarr CL (2020). A Vibrio cholerae core genome multilocus sequence typing scheme to facilitate the epidemiological study of cholera. J Bacteriol.

[11] Goering RV (2010). Pulsed field gel electrophoresis: a review of application and interpretation in the molecular epidemiology of infectious disease. Infect Genet Evol.

[12] Lukinmaa S, Nakari UM, Eklund M, Siitonen A (2004). Application of molecular genetic methods in diagnostics and epidemiology of food-borne bacterial pathogens. Apmis.

[13] Teh CSJ, Chua KH, Thong KL (2011). Genetic variation analysis of Vibrio cholerae using multilocus sequencing typing and multi-virulence locus sequencing typing. Infect Genet Evol.

[14] Pavón ABI, Maiden MC (2009). Multilocus sequence typing.

[15] Chan M-S, Maiden MC, Spratt BG (2001). Database-driven multi locus sequence typing (MLST) of bacterial pathogens. Bioinformatics.

[16] Urwin R, Maiden MC (2003). Multi-locus sequence typing: a tool for global epidemiology. Trends Microbiol.

[17] Bakhshi B, Mahmoudi-Aznaveh A, Salimi-Khorashad A (2015). Clonal dissemination of a single Vibrio cholerae O1 biotype El Tor strain in Sistan-Baluchestan province of Iran during 2013. Curr Microbiol.

[18] Bakhshi B, Boustanshenas M, Mahmoudi-aznaveh A (2014). Emergence of V ibrio cholerae O 1 classical biotype in 2012 in I ran. Lett Appl Microbiol.

[19] Stavric S, Bachanan B. The isolation and identification of V. cholerae O1 and non-O1 from foods. Government of Canada, Health Protection Branch, Ottawa, Polyscience Publication, MFLP-72, Canada. 1995.

[20] Choopun N, Louis V, Huq A, Colwell RR (2002). Simple procedure for rapid identification of Vibrio cholerae from the aquatic environment. Appl Environ Microbiol.

[21] Control CfD, Prevention. Laboratory methods for the diagnosis of Vibrio cholerae. Atlanta, GA. 1994;500:38–67.

[22] Chun J, Rivera IN, Colwell RR (2002). Analysis of 16S–23S rRNA intergenic spacer of Vibrio cholerae and Vibrio mimicus for detection of these species.

[23] CLSI C. Methods for antimicrobial dilution and disk susceptibility testing of infrequently isolated or fastidious bacteria; approved guideline. Clinical and Laboratory Standards Institute (M45eA2). 2010.

[24] Weinstein MP, Limbago B, Patel J, Mathers A, Campeau S, Mazzulli T, et al. M100 performance standards for antimicrobial susceptibility testing. CLSI; 2018.

[25] vibrio-pfge-protocol [Available from: https://www.cdc.gov/pulsenet/pdf/vibrio-pfge-protocol-508c.pdf.

[26] Tenover F, Arbeit R, Goering R, the Molecular Typing Working Group of the Society for Healthcare Epidemiology of America (1997). How to select and interpret molecular strain typing methods for epidemiological studies of bacterial infections: a review for healthcare epidemiologists. Infect Control Hosp Epidemiol.

[27] Amin Marashi SM, Bakhshi B, Imani Fooladi AA, Tavakoli A, Sharifnia A, Pourshafie MR (2012). Quantitative expression of cholera toxin mRNA in Vibrio cholerae isolates with different CTX cassette arrangements. J Med Microbiol.

[28] Criscuolo A. AlienTrimmer User Guide.

[29] Liu Y, Schröder J, Schmidt B (2013). Musket: a multistage k-mer spectrum-based error corrector for Illumina sequence data. Bioinformatics.

[30] Prjibelski A, Antipov D, Meleshko D, Lapidus A, Korobeynikov A (2020). Using SPAdes de novo assembler. Curr Protoc Bioinformatics.

[31] Hur Y, Chalita M, Ha S-m, Baek I, Chun J (2019). VCGIDB: a database and web resource for the genomic islands from Vibrio Cholerae. Pathogens.

[32] Zankari E, Hasman H, Cosentino S, Vestergaard M, Rasmussen S, Lund O (2012). Identification of acquired antimicrobial resistance genes. J Antimicrob Chemother.

[33] Carattoli A, Zankari E, García-Fernández A, Voldby Larsen M, Lund O, Villa L (2014). In silico detection and typing of plasmids using PlasmidFinder and plasmid multilocus sequence typing. Antimicrob Agents Chemother.

[34] Herrera CM, Crofts AA, Henderson JC, Pingali SC, Davies BW, Trent MS (2014). The Vibrio cholerae VprA-VprB two-component system controls virulence through endotoxin modification. MBio.

[35] Matson JS, Livny J, DiRita VJ (2017). A putative Vibrio cholerae two-component system controls a conserved periplasmic protein in response to the antimicrobial peptide polymyxin B. PLoS ONE.

[36] Rowe-Magnus DA, Guerout AM, Mazel D (2002). Bacterial resistance evolution by recruitment of super-integron gene cassettes. Mol Microbiol.

[37] Weill F-X, Domman D, Njamkepo E, Tarr C, Rauzier J, Fawal N (2017). Genomic history of the seventh pandemic of cholera in Africa. Science.

[38] Smith AM, Weill F-X, Njamkepo E, Ngomane HM, Ramalwa N, Sekwadi P (2021). Emergence of Vibrio cholerae o1 sequence type 75, South Africa, 2018–2020. Emerg Infect Dis.

[39] Fonseca ÉL, dos Santos FF, Vieira VV, Vicente AC (2008). New qnr gene cassettes associated with superintegron repeats in Vibrio cholerae O1. Emerg Infect Dis.

[40] Zhou Y-Y, Ma L-Y, Yu L, Lu X, Liang W-L, Kan B (2023). Quinolone Resistance Genes and Their Contribution to Resistance in Vibrio cholerae Serogroup O139. Antibiotics.

[41] Hajia M, Sohrabi A (2019). In silico characteristics for re-emerging possibility of Vibrio cholerae genotypes in Iran. New Microbes New Infect.

[42] Mafi M, Goya MM, Hajia M (2016). A five-year study on the epidemiological approaches to cholera in Iran. Caspian J Intern Med.

[43] Liao F, Mo Z, Chen M, Pang B, Fu X, Xu W (2018). Comparison and evaluation of the molecular typing methods for toxigenic Vibrio cholerae in Southwest China. Front Microbiol.

[44] Vilela FP, Falcão JP (2021). Analysis of the antimicrobial resistance gene frequency in whole-genome sequenced Vibrio from Latin American countries. J Med Microbiol.

[45] Breurec S, Franck T, Njamkepo E, Mbecko J-R, Rauzier J, Sanke-Waïgana H (2021). Seventh pandemic vibrio cholerae O1 sublineages, Central African Republic. Emerg Infect Dis.

[46] Greig DR, Schaefer U, Octavia S, Hunter E, Chattaway MA, Dallman TJ (2018). Evaluation of whole-genome sequencing for identification and typing of Vibrio cholerae. J Clin Microbiol.

[47] Luo Y, Octavia S, Jin D, Ye J, Miao Z, Jiang T (2016). US Gulf-like toxigenic O1 Vibrio cholerae causing sporadic cholera outbreaks in China. J Infect.

[48] Okada K, Roobthaisong A, Swaddiwudhipong W, Hamada S, Chantaroj S (2013). Vibrio cholerae O1 isolate with novel genetic background, Thailand-Myanmar. Emerg Infect Dis.

