# Supplementary material for: Genetic approach toward linkage of Iran 2012–2016 cholera outbreaks with 7th pandemic Vibrio cholerae
Source: BMC Microbiol. 2024 Jan 22;24:33. doi: 10.1186/s12866-024-03185-9 (PMC10801964; doi:10.1186/s12866-024-03185-9)
Supplement: Supplementary file 1 — Additional file 1. [file 12866_2024_3185_MOESM1_ESM.docx]

1)


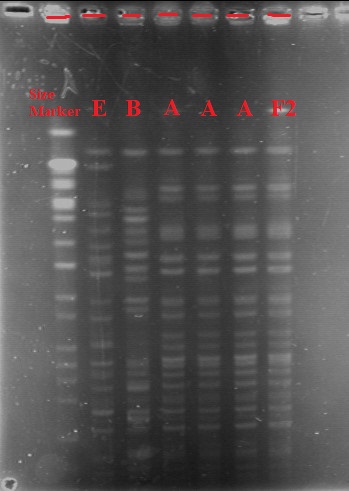


**2)**


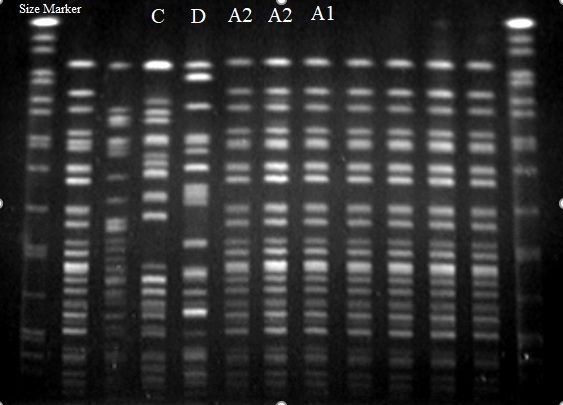


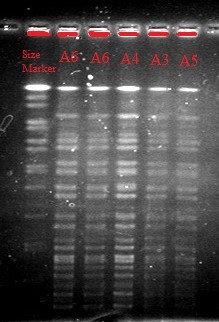


**3)**

**4)**


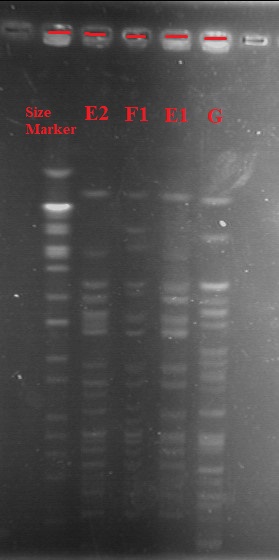


**Supplementary Figure A. Pulsed-field gel electrophoresis (PFGE) gel images of *V.cholerae* strains restricted with *NotI*.**

**1)** Pulsotypes of *V.cholerae* strains from left to right: size marker, E, B, A, A, A, F2

**2)** Pulsotypes of *V.cholerae* strains from left to right: 1, size marker; C, D, A2, A2, A1

* Full length membrane and adequate length of current gel image were not available.

**3)** Pulsotypes of *V.cholerae* strains from left to right: size marker, A6, A6, A4, A3, A5

**4)** Pulsotypes of *V.cholerae* strains from left to right: size marker, E2, F1, E1, G

** The samples derive from the same experiment and that gels were processed in parallel.

Blots were not cut prior to hybridisation with antibodies.
